# Music to Make Your Mouth Water? Assessing the Potential Influence of Sour Music on Salivation

**DOI:** 10.3389/fpsyg.2017.00638

**Published:** 2017-04-26

**Authors:** Qian J. Wang, Klemens Knoeferle, Charles Spence

**Affiliations:** ^1^Crossmodal Research Laboratory, Department of Experimental Psychology, Oxford UniversityOxford, UK; ^2^Department of Marketing, BI Norwegian Business SchoolOslo, Norway

**Keywords:** salivation, crossmodal correspondences, taste perception, audiovisual stimuli, physiological response

## Abstract

People robustly associate various sound attributes with specific smells/tastes, and soundtracks that are associated with specific tastes can influence people’s evaluation of the taste of food and drink. However, it is currently unknown whether such soundtracks directly impact the eating experience via physiological changes (an embodiment account), or whether they act at a higher cognitive level, or both. The present research assessed a version of the embodiment account, where a soundtrack associated with sourness is hypothesized to induce a physiological response in the listener by increasing salivary flow. Salivation was measured while participants were exposed to three different experimental conditions – a sour soundtrack, a muted lemon video showing a man eating a lemon, and a silent baseline condition. The results revealed that salivation during the lemon video condition was significantly greater than in the sour soundtrack and baseline conditions. However, contrary to our hypothesis, there was no significant difference between salivation levels in the sour soundtrack compared to the baseline condition. These results are discussed in terms of potential mechanisms underlying the auditory modulation of taste perception/evaluation.

## Introduction

Recently, it has been demonstrated that people tend to robustly associate attributes of sound with specific olfactory (i.e., smell) and gustatory (i.e., taste) stimuli. For instance, consonant harmonies and legato musical articulation tends to be associated with sweetness, while dissonant harmonies and staccato articulation tends to be associated with sourness instead (e.g., Mesz et al., 2011; Wang and Spence, 2016). In addition, both sweet and sour tastes are mapped to high pitch whereas bitter tastes are mapped to low pitch (Crisinel and Spence, 2010; Mesz et al., 2011; Knoeferle et al., 2015; Wang et al., 2016). Furthermore, these sound-taste correspondences can affect people’s evaluation of the taste/flavor of foods. For example, ratings of juice samples on a sweet–sour scale varied significantly depending on the consonance/dissonance levels of the background musical composition that people heard in one recent study (Wang and Spence, 2016). However, what is currently still unclear is whether these changes in taste evaluation occur at a low level (i.e., by directly influencing sensory experience), and/or at a higher level, such as by priming people’s expectations or by biasing their self-reported taste ratings.

A possible low level hypothesis, investigated in the present study, is an embodied account, whereby people might associate certain soundtracks with certain tastes because the soundtracks induce a similar physiological response in the listener as ingesting foods having that taste property. More specifically, we hypothezise that people might associate a soundtrack with sourness because the soundtrack, much like sour foods, can increase the listeners’ salivary flow. Salivation is a non-conscious physiological process controlled by the autonomic nervous system, which aids in the digestion process and can influence the perception of tastants in the mouth (see Spence, 2011, for a review). Salivation can also be induced by conditioned reflexes, such as seeing or smelling appetizing foods (Wooley and Wooley, 1973; Krishna et al., 2014), or even by a goal-driven material reward (Gal, 2012). Previous research has shown that while looking at a lemon does not increase salivation (Kerr, 1961; Shannon et al., 1974), sniffing or slicing lemons does (e.g., Pangborn, 1968; Pangborn et al., 1979). Looking at a video of someone else eating a lemon has also been shown to induce salivation (Hagenmuller et al., 2014). Therefore, to check the validity of our methodology, we used a video of a man eating a lemon (henceforth referred to as “lemon video condition,” which should increase salivation (thus demonstrating the sensitivity of our measurement technique).

Furthermore, participants from our previous studies have occasionally commented on the “mouth-watering” effect of high-pitched and dissonant soundtracks which were composed to correspond to sourness. Previously, it has been shown that music can influence the composition of salivation (Suda et al., 2008), with major mode music reducing salivary cortisol levels as compared to minor mode music. While there are no studies relating salivary cortisol levels with effects on taste perception, those who exhibit higher cortisol level increases due to stress also tend to consume more foods, including more sweet foods, as compared to those who experience lesser cortisol level changes (Epel et al., 2001). Spoken food words have also been shown to increase salivation compared to non-food words (Staats and Hammond, 1972). Based on these results, we would expect that a putatively sour soundtrack might enhance the level of salivation in the listener. This might especially be the case if the listener explicitly associates the soundtrack with the idea of sourness (i.e., if they were to match the soundtrack to sourness in a forced-choice task with multiple taste words as options, say).

To test this embodiment account hypothesis, a study was designed to measure levels of salivation under different audio/video conditions. Several methods of saliva collection have been used over the years, including the absorption of saliva by dental cotton rolls, measuring the frequency of swallows, or the electrophysiological measurement of parotid gland activity (Nederkoorn et al., 2001). The method of cotton-roll collection is used in the current study, as it has been shown to provide a reliable, sensitive, and straightforward means of measuring salivary flow (White, 1977).

## Materials and Methods

### Participants

Thirty six participants (22 women, 14 men) aged between 18 and 49 years (*M* = 23.1, *SD* = 6.6) took part in the study. The participants reported no hearing impairments. The participants were recruited from the Oxford Psychology Research Participant Database and the Experimental Psychology Research Participation Scheme. The study was carried out in accordance with the recommendations of the Central University Research Ethics Committee of Oxford University, with the written informed consent of all subjects. All subjects gave written informed consent in accordance with the Declaration of Helsinki. The protocol was approved by the Central University Research Ethics Committee of Oxford University (R47262_RE001).

### Audio/Video Stimuli

Three audio/video stimuli were used. As a sour soundtrack, we used a high-pitched and dissonant soundtrack composed by Bruno Mesz that has been shown to reliably correspond to sourness based on previous studies (Kontukoski et al., 2015). In fact, Wang et al. (2015) compared seven soundtracks that have been designed to correspond to the experience of tasting sourness. In the study, the soundtrack by Mesz was labeled as sour, as opposed to any other basic taste, by the largest number of participants^1^ (58/100). A silent video of a man eating a lemon was used as an additional condition (the “lemon video condition”) to verify the validity of the saliva measurement methodology used here, since it has previously been shown to elicit salivation (Hagenmuller et al., 2014). The specific 60-s segment of the video can be viewed at https://www.youtube.com/watch?v=5FfHSUVBIdw#t=63s. Finally, a silent condition (via a soundtrack with the commands “start” and “stop” separated over a 60 s interval) was included as a baseline saliva measure. All three conditions were 60 s long. The sour soundtrack and baseline conditions were accompanied by a visual target (+) for participants to focus on while listening to the soundtracks.

### Procedure

The experiment was conducted at the Crossmodal Research Laboratory at the University of Oxford. Participants were seated at a table in front of a computer monitor with a keyboard, mouse, and headphones in an experimental booth. On the side table were six small plates each with three 8 mm dental cotton rolls, a cup of water, and a napkin.

On each trial, the participants were instructed to place three cotton dental rolls in their mouth, two buccally and one under the tongue, then immediately start playing the soundtrack or video. Once the soundtrack or video had finished, the participants were asked to remove the cotton rolls immediately, place them back on the plate, and hand them to the experimenter. Each trial lasted for 60 s, and the participants were given a 5 min recovery period between trials. Each condition was repeated twice (not necessarily successively), thus giving rise to a total of six trials. The order in which the trials were presented was determined using a Williams Design Latin Square in order to minimize first order carryover effects between trials. The cotton rolls were disposed of immediately after weighing.

After the saliva collection trials, the participants rated which basic taste (sweet, sour, bitter, salty) the soundtrack best matched with, and more specifically, how well the sour soundtrack matched with sweet, sour, and bitter tastes on three 1–7 scales (1 = does not match at all, 7 = matches very well). They also reported their age and gender.

The study lasted for approximately 35–40 min. The participants were paid £6 or awarded with course credit for taking part.

### Data Analysis

To determine the level of induced salivation, the cotton rolls were weighed before and immediately after each trial, on a balance with 0.0001 g precision. The difference between the two weights was used as the amount of induced saliva. The mean weight of induced salivation was then calculated for each condition and each participant.

A repeated measures analysis of variance (RM-ANOVA) was conducted with the factor ‘experimental condition’ (sour soundtrack, lemon video, silence). In addition, the model included participants’ rating of whether they matched the soundtrack with sourness as a between-participants variable, and the interaction term of the experimental condition and the between-participants variable.

Furthermore, we calculated the % increase of salivation for each participant while listening to the sour soundtrack as compared to the silence condition. We then calculated Pearson’s correlation coefficient between this % increase and how much the participant matched the soundtrack to sourness, in order to determine if sensitivity to the soundtrack’s intended taste representation influenced their level of salivation.

## Results

**FIGURE 1:**
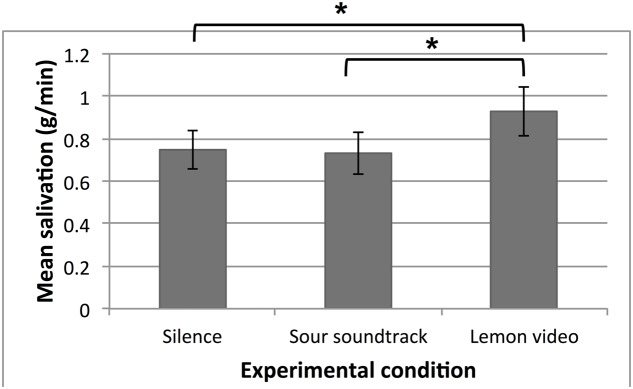
**Participants’ mean salivation (g/min) in all three 60-s experimental conditions.** Error bars indicate standard errors. Asterisks denote statistical significance (^∗^*p* < 0.05).

The average salivation level for each experimental condition is shown in **Figure 1**. RM-ANOVA with Huynh-Feldt corrections revealed a significant main effect of experimental condition on salivation [*F*(1.82,61.77) = 6.85, *p* = 0.003, η^2^ = 0.17], but no significant main effect of soundtrack-sourness match [*F*(1,34) < 0.000, *p* = 0.99] and no interaction effect between the two [*F*(2,68) = 0.17, *p* = 0.84]. More specifically, pairwise comparisons with Bonferroni corrections revealed that more salivation was measured during the lemon video condition (*M*_video_ = 0.93 g, *SD* = 0.68) as compared to the silent condition (*M*_silence_ = 0.74 g, *SD* = 0.53, *p* = 0.006) or the sour soundtrack condition (*M*_soundtrack_ = 0.74 g, *SD* = 0.58, *p* = 0.029). The sour soundtrack condition, however, did not significantly differ from the silent baseline condition (*p* = 1.00). This result does not support our hypothesis, which stated that listening to the sour soundtrack would induce increased salivation compared to the baseline condition.

**FIGURE 2:**
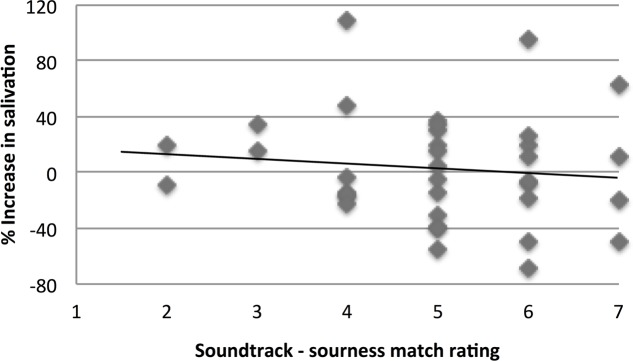
**Correlation plot between the % increase in salivation between the sour soundtrack condition as compared to the silent baseline condition, and the soundtrack-taste match rating between the sour soundtrack and sourness (1 = does not match at all, 7 = matches very well).** The black line indicates line of best fit. There is no significant correlation (*r*_36_ = –0.11, *p* = 0.51).

Moreover, there was no significant correlation between % increase of salivation while listening to the sour soundtrack as compared to silence, and the rating of how much the sour soundtrack was matched to sourness (*r*_36_ = -0.11, *p* = 0.51, see **Figure 2** for a correlation plot). In other words, the extent to which someone matched the sour soundtrack to sourness is not related to any increase in the amount of salivation while listening to the sour soundtrack.

## Discussion

The results of the present study reveal that, as reported previously, watching a video of someone eating a lemon induces increased salivation as compared to the baseline condition, i.e., silently looking at a fixation cross. This replication of previous results (Hagenmuller et al., 2014; see Spence, 2011, for a review) validates our methodology of using dental rolls to measure salivation. On average, the lemon video condition increased salivation by 0.18 g as compared to the baseline condition. This is similar to the findings reported by Hagenmuller et al. (2014) where a different lemon video increased salivation by approximately 0.25 g over a 60-s interval.

However, we found no evidence that listening to the sour soundtrack increased salivation in our participants compared to the silent baseline condition. This result is especially telling given that we used the most effective – in terms of being associated with sourness – soundtrack that has been tested to date (Wang et al., 2015). Perhaps auditory stimulation is simply not sufficient to evoke a physiological response; this may be in-line with previous research which showed that while looking at lemons does not induce increased salivation (Kerr, 1961; Shannon et al., 1974), smelling or slicing lemons does (Pangborn, 1968; Pangborn et al., 1979). Therefore, like a visual representation of lemons, the soundtrack alone might not evoke a strong enough representation of sourness to stimulate a physiological response. In line with this suggestion, it has been theorized that while sound may influence the overall eating experience, it has a relatively weak contribution (Mroczko-Wąsowicz, 2016). The resulting percept is consciously decomposable into its component unisensory parts (e.g., hearing the sound of crunching makes potato crisps more crunchy, but it is easy to separate the sound of mastication from the flavor of the potato crisp). In contrast, olfaction has a strong (what Mroczko-Wąsowicz termed “constitutive”) contribution which binds with information from the tongue to form a unified flavor perception (Rozin, 1982; Spence et al., 2015).

In this study, we hypothesized that people might associate a soundtrack with sourness because the soundtrack, much like sour foods, could potentially increase the listeners’ salivary flow. The fact that no increase in salivation was found between the soundtrack condition and the baseline condition allows us to conclude that, contrary to our initial hypothesis, we failed to observe any enhancement of salivation due to music, even when participants associated the soundtrack with sourness. Therefore, there is no evidence for this particular version of the embodiment hypothesis in the present study. Interestingly, as the lemon video did, in fact, evoke increased salivation, it implies that top–down effects are at work, where an understanding of the visual scene could trigger a mental imagery of eating a lemon, which would then produce the physiological response observed (Jenkins and Dawes, 1966).

Going back to the different high and low level mechanisms proposed in the “Introduction” section, it is worth enumerating here which other pathways could be underlying the taste modulation effects by putatively sour soundtracks, such as reported by Wang and Spence (2016). Besides physiological influences, another bottom–up mechanism could involve attentional capture. According to this view, auditory features might automatically focus our attention on taste elements in the food that crossmodally correspond to those features. This focused attention could then enhance the salience of the attended feature in a mixture (in this case, sour tastes in a food/drink), relatively to when the same feature is unattended (Driver, 2001; Spence, 2014). In terms of top–down influences, associating soundtracks with sourness might prime people’s sensory expectations of sourness in the food that they are about to consume, which could then go on to influence the perceptual experience (see Deliza and MacFie, 1996; Piqueras-Fiszman and Spence, 2015, for reviews). Finally, it is worth considering the possibility that the sour soundtracks might act only to alter participants’ self-reported ratings without having a genuine perceptual effect.

Another intriguing question from the present study is just where exactly such crossmodal associations between sourness and sounds might come from. One hypothesis of sound-taste correspondences – specifically that between auditory pitch and taste^2^ – is that the correspondence originates in innate stereotypical orofacial gestures that people make in response to ingesting different tastes (Knöferle and Spence, 2012; Spence, 2012; Bredie et al., 2014). Babies protrude their tongue out and up in response to pleasant tastes such as sweetness (Rosenstein and Oster, 1988; Steiner et al., 2001). This in turn produces a high vowel sound when air is exhaled (Ladefoged and Johnson, 2011). In contrast, the tongue goes out and down in response to unpleasant tastes (e.g., bitterness), which then produces a low vowel sound upon exhalation. Unlike in the case of sweetness and bitterness, however, this does not account for the fact that sourness, which is traditionally characterized as aversive, corresponds to high pitch.

An alternative hypothesis is based on emotion mediation, which seeks to explain the association between sourness and auditory attributes such as fast tempo, high pitch, and high levels of harmonic dissonance. The experience of ingesting a sour taste is associated with higher levels of arousal as compared to the other tastes (Wang et al., 2016). Similarly, the experience of listening to these auditory parameters is also associated with high arousal levels (e.g., Blumstein et al., 2010; Van der Zwaag et al., 2011; Wang et al., 2016). Therefore, the correspondence between sourness and sound might be linked to their similar associations with high arousal states.

While the present study does not support a physiological link between sound and sourness, it is still possible that music, in conjunction with other sensory stimuli, might act to enhance physiological responses to food/drinks. An interesting future study, for instance, could compare the lemon video condition with a combined lemon video plus sour soundtrack condition, to assess if music might act to further enhance salivation.

## Author Contributions

QW and CS designed the study. QW collected the data and designed the experimental stimuli. QW and KK performed data analysis. All authors participated in manuscript preparation and all authors read and approved the final manuscript.

## Conflict of Interest Statement

The authors declare that the research was conducted in the absence of any commercial or financial relationships that could be construed as a potential conflict of interest. The reviewer MB and handling Editor declared their shared affiliation, and the handling Editor states that the process nevertheless met the standards of a fair and objective review.

## Footnotes

1 Note that, if participants chose tastes at random, the soundtrack would be labeled as sour by 25/100 people.

2 It has been demonstrated that sweetness and sourness corresponds to relatively higher pitch whereas bitterness corresponds to relatively lower pitch (Crisinel and Spence, 2010; Wang et al., 2016).
